# Application of N-Butyl-2-Cyanoacrylate for the Treatment of Comminuted Fractures in the Anterior Wall of the Maxillary Sinus: A Prospective Clinical Study

**DOI:** 10.7759/cureus.35487

**Published:** 2023-02-26

**Authors:** Abdul Hafeez. A, Sameeya Furmeen. S, Duraisamy Durairaj, M. Arulmozhi Rajasekaran, Davidson Rajiah

**Affiliations:** 1 Department of Oral and Maxillofacial Surgery, Tamil Nadu Government Dental College and Hospital, Chennai, IND; 2 Dentistry and Oral and Maxillofacial Surgery, All India Institute of Medical Sciences, Raipur, IND; 3 Department of Community Medicine, Stanley Government Medical College, Chennai, IND; 4 Department of Oral and Maxillofacial Surgery, Adhiparasakthi Dental College and Hospital, Chennai, IND

**Keywords:** bone adhesive, trauma management in maxillofacial region, n-butyl-2-cyanoacrylate, maxillary sinus, comminuted fracture

## Abstract

Objective

The purpose of the study was to assess the clinical outcome of patients by using n-butyl-2-cyanoacrylate in the management of comminuted fracture segments of the anterior wall of the maxillary sinus in the zygomatico-maxillo-facial complex region.

Material and methods

A prospective study was done at a tertiary care teaching institute in India with a study population of ten patients in a single group. The method of recruitment was a convenient sampling method. Out of all the study subjects, three patients had isolated maxillary sinus wall fractures, whereas the remaining seven had other associated facial fractures that required stable fixation with mini plates. The comminuted fractures of the anterior wall of the maxillary sinus were carefully reduced through an intra-oral approach, and n-butyl-2-cyanoacrylate was applied over the edges of fractured segments. The segments were left undisturbed for one minute and closed with a 3-0 vicryl. The outcome variables, namely bone alignment visualised through computed tomography (CT) scan postoperatively, paresthesia or hypoesthesia of the infraorbital nerve, postoperative infection, and wound dehiscence, were noted at one-week, one-month, three-month, and six-month intervals. Data were analysed using the Chi-square test.

Results

Among all patients, seven had satisfactory bone alignment. A total of seven patients recovered from hypoesthesia of the infraorbital nerve. The association of bone alignment with hypoesthesia or paresthesia revealed a highly significant p-value (0.002) using the Chi-square test. Also, an association between postoperative infection and wound dehiscence showed substantial results with a p-value less than 0.05.

Conclusion

Good bone alignment was seen postoperatively in 70% of patients. The cyanoacrylate used had no adverse reactions, and its application was restricted to non-load-bearing areas in this study. Further studies with a higher level of evidence and a larger sample size are needed to validate the use of adhesives for bone fixation in other regions of the face.

## Introduction

Facial fractures can range in complexity from a straightforward hairline fracture to a challenging pan-facial fracture. The comminuted fractures of the facial skeleton are one of the most challenging fractures to rehabilitate. The comminuted fractures of load-bearing regions in the face, such as the mandible or the region of the zygomatico-maxillary buttress, are supposed to be reduced and fixed with a thicker profile plating system. However, the anterior wall of the maxillary sinus, a common location for a clinician to detect comminuted fractures, is usually left untreated. This is due to the fact that the bony anterior wall of the maxillary sinus is relatively thin and hollow inside and does not constitute part of the vertical or horizontal pillars to transmit occlusal forces. A large number of surgeons who typically treat facial fractures end up disregarding comminuted fractures of the maxillary sinus anterior wall. These fragments could be reconstructed in accordance with the natural anatomy of the face thanks to the development of several novel biomaterials. One such biomaterial is n-butyl-2-cyanoacrylate. In craniofacial surgery, biocompatible glues have already been used successfully to heal corneal thinning, perforations, and retinal tears [1]. They are also used as hemostatic agents and embolic substances. Cyanoacrylates can function in a moist environment and have been proven to have strong biomechanical strength [2]. It is also used as a soft tissue adhesive in maxillofacial surgery for intra-oral mucosal closures as well as skin closures [3]. The purpose of the study was to assess the clinical outcome of patients by using n-butyl-2-cyanoacrylate in the management of comminuted fracture segments of the anterior wall of the maxillary sinus in the zygomatico-maxillary complex region. The primary objective of the study was to evaluate the bony alignment of the sinus wall, whereas the secondary objective was to evaluate the hypoesthesia and paresthesia of cutaneous sensation supplied by the infraorbital nerve and to determine whether any statistically significant association was present between bony alignment and paresthesia of the nerve.

## Materials and methods

The following study was done at the Department of Oral and Maxillofacial Surgery of the Tamil Nadu Government Dental College and Hospital, Chennai, India. This study was conducted prospectively after obtaining approval from the institutional ethics committee (0420/DE/2016). The study was conducted for a little more than one year, from 2017 to mid-2018. A total of ten patients were included in the study and were followed up for six months postoperatively. There was no loss of follow-up.

Inclusion and exclusion criteria

All patients who reported to the department were screened for comminuted fractures involving the anterior wall of the maxillary sinus using a convenient sampling method. Malunited fracture segments with trauma older than 15 days were not considered in the study. Other exclusion criteria were cases with a history of tobacco smoking or consumption of smokeless tobacco, an infected fracture site, and patients with immunocompromised diseases or other systemic illnesses that can impair tissue healing.

Patients older than 18 years of age who met the above criteria were taken up for the study with prior informed consent. The study protocol was explained to all the patients before the procedure. A complete medical history was taken, and a detailed clinical examination was done. It was followed by routine blood investigations as part of the workup for the surgery. Any abnormalities in investigations suspicious of systemic illness were excluded from the study.

Primary and secondary outcome variables

The study's primary outcome variable was to evaluate and compare bone integrity and alignment in the comminuted fracture site pre-operatively and postoperatively using CT scans (axial sections). The secondary outcome variable was to evaluate hypoesthesia of the cutaneous regions innervated by terminal branches of the maxillary nerve, particularly the infra-orbital nerve. Other outcome variables noted were post-operative infection, any adverse reaction, and wound dehiscence.

The radiological assessment with a CT scan was done postoperatively at one week. The clinical evaluation was done on the first postoperative day and at the end of one-week, one-month, three-month, and six-month intervals.

Surgical procedure

**Figure 1:**
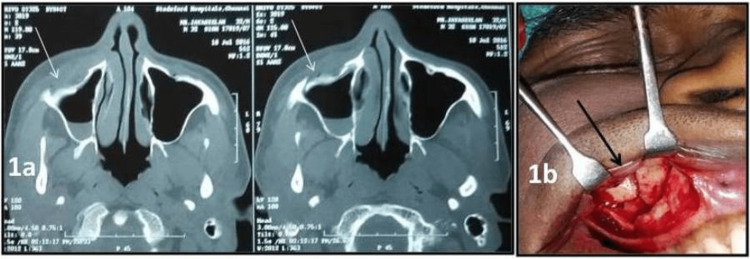
1a: comminuted fracture of the anterior wall of the maxillary sinus as evident in the CT scan axial section (preoperative); 1b: clinical picture of fractured segments

Under stringent aseptic conditions and local anaesthesia, submucosal infiltration using lignocaine with adrenaline (1:80,000) was used to treat all patients. A standard maxillary vestibular incision was placed intraorally, and a full-thickness mucoperiosteal flap was raised until the comminuted fracture of the anterior wall of the maxillary sinus was exposed. If there was clinically evident compression of the infraorbital nerve backed up by paraesthesia, then the nerve was released from the foramen. Once the surgical site is exposed, the fracture segments are carefully elevated or reduced with the use of microinstruments (Figures 1A, 1B).

**Figure 2:**
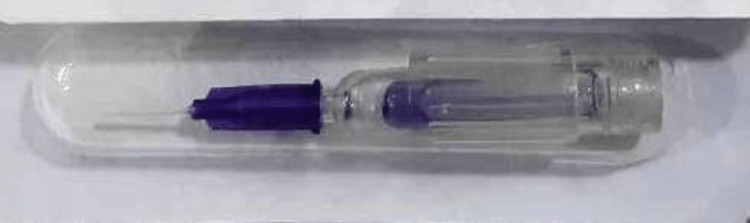
The adhesive n-butyl-2-cyanoacrylate, as explained in a glass ampule inside a plastic container with a soft microdelivery tip

The n-butyl-2-cyanoacrylate adhesive used was TRUSEAL, manufactured by Reevax Pharmaceuticals, India. It's available in a single-use sterile plastic container with a soft microneedle delivery system and has a built-in crushable glass ampule containing 0.25 cc of the active ingredient in a liquid state. The product is prevented from hardening owing to ambient moisture by the crushable ampule mechanism. This ensures that the product stays liquid, remains active and is effective at the time of application (Figure 2).

**Figure 3:**
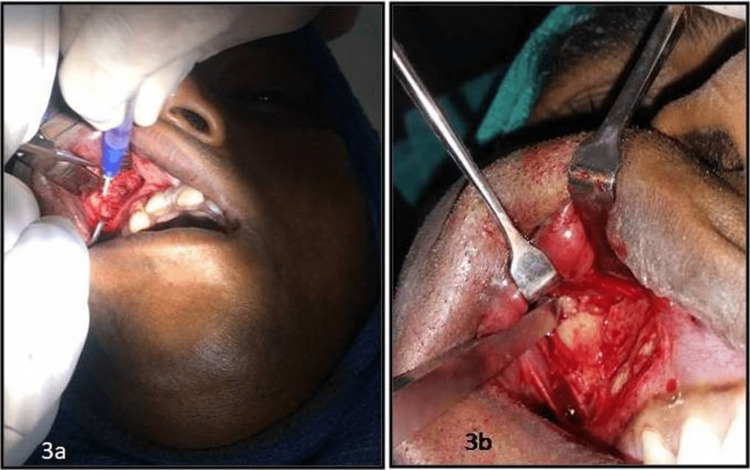
3a: application of the cyanoacrylate adhesive; 3b: reduced fracture segments after its application

The glass ampule is crushed with the help of a haemostat and the material, as it gets in contact with air moisture, starts to solidify in three to five seconds and finishes its solidification reaction in 60 to 90 seconds. Accidental overapplication is minimized, resulting in faster healing and less tissue damage from the heat of polymerization. The liquid adhesive is carefully applied onto the edges of the comminuted fractured segments, which were reduced and held in place (Figures 3a, 3b).

**Figure 4:**
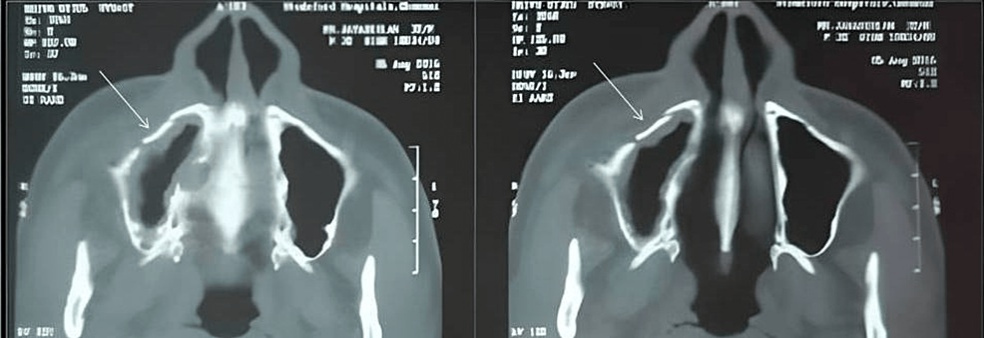
Reduction of fractured segments, as seen in the axial section of the CT scan (postoperative)

After adequate time has passed, the surgical site was irrigated with a copious volume of betadine and saline, and closure was done with 3-0 vicryl. Antibiotics and analgesics (amoxicillin 500 mg, three times daily; metronidazole 400 mg, three times daily; and aceclofenac 100 mg, twice daily) were given for five days, along with xylometazoline 0.1% nasal drops for any nasal blockage. Patients were advised not to lie in a lateral recumbent position on the operated side for three days. They were also advised to maintain good oral hygiene. A postoperative CT scan was taken in one week to evaluate the reduction in axial sections (Figure 4).

Postoperative assessment and follow-up

All the study parameters, namely bone alignment in the CT scan, hypoesthesia or paresthesia, postoperative infection, adverse reaction, and wound dehiscence, were noted as dichotomous variables. Bone alignment was classified as satisfactory or unsatisfactory. Other parameters were classified as present or absent.

The bone alignment was assessed by repeating the CT scan after one week and comparing the preoperative and postoperative images of the maxillary sinus anterior wall in axial sections. Those fractures that showed good alignment and reduction were classified as satisfactory, and those that had improper alignment to the satisfaction of the operating and assisting surgeons were classified as unsatisfactory.

Hypoesthesia or paresthesia of the terminal branches of the maxillary nerve, involving the infraorbital nerve and anterior superior alveolar nerve, was characterised by numbness in the involved side of the upper lip and the gingiva of the upper teeth. This parameter was evaluated objectively preoperatively, immediately the next day of the postoperative period, and was also assessed in the consecutive follow-up periods of one week, one month, three months, and six months. This was done with the help of a two-point discrimination test. During these interval periods, other variables like wound dehiscence, signs of infection, and adverse reactions were also noted.

Data analysis

The data collected was analysed using IBM's Statistical Package for Social Sciences (SPSS) software, version 21. The frequency distribution of age, gender, mode of injury, and associated fracture or isolated fracture was done. Results obtained were analysed using the Chi-square test, and degrees of association for each parameter with other parameters were assessed. The p-value was obtained and levels of significance were noted. A p-value less than 0.05 was considered to be statistically significant.

## Results

This prospective study had a total of ten subjects at the end of the follow-up period. The participants were aged between 19 and 42 years, with the mean age being 29 years and a standard deviation of 7.28. Among the ten participants, eight were males and two were females. Among the study population, three patients had isolated anterior maxillary sinus wall fractures and seven had associated other facial fractures that required open reduction and stable internal fixation with mini plates and screws. The aetiologies of injuries were different. Four patients had road traffic accidents (RTAs), three had domestic violence or assault injuries, two had sports injuries, and one suffered from a self-reported fall.

Bone alignment

**Figure 5:**
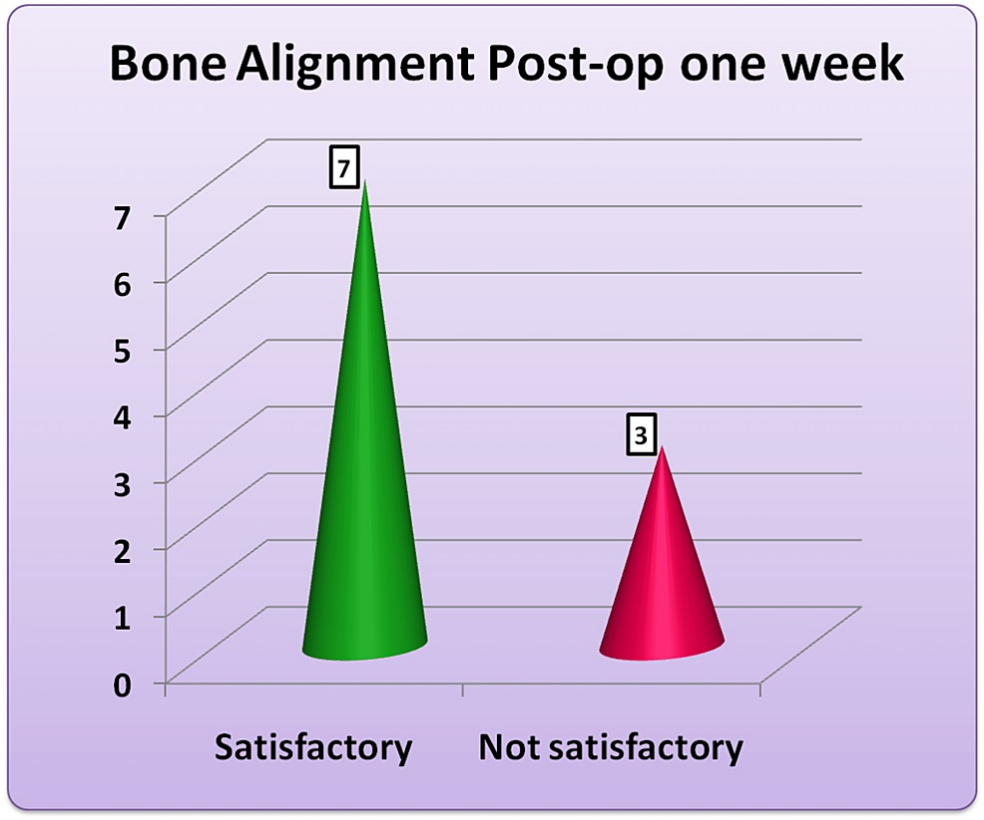
The frequency distribution of bone alignment during the postoperative follow-up at one week

Among all patients, seven patients had a satisfactory bone alignment in the postoperative CT scan (axial sections), while the remaining three had an unsatisfactory alignment (Figure 5).

Hypoesthesia or paresthesia

**Figure 6:**
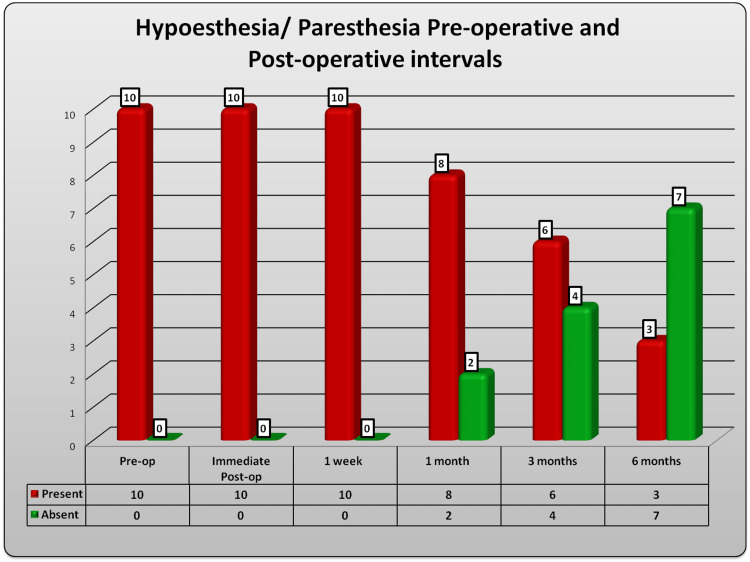
Hypoesthesia: preoperative and postoperative comparison

All the patients had preoperative hypoesthesia or paresthesia of cutaneous innervations by the terminal branch of the maxillary nerve, namely the infraorbital nerve. All patients had the continued presence of hypoesthesia or paresthesia until one week postoperatively. In the first month of follow-up, two patients recovered from numbness. In the third month, the number had doubled to four. In the sixth-month follow-up, only three patients had post-operative hypoesthesia or paresthesia, while seven showed signs of recovery. It is to be noted that the three patients for whom hypoesthesia did not get corrected had an unsatisfactory bone alignment in the postoperative CT scan (Figure 6).

Postoperative infection and wound dehiscence

Among the ten patients, only two showed signs and symptoms of postoperative infection. Out of the two patients, one had the characteristics of maxillary sinusitis, which are foul-smelling breaths and pain while bending down. The patient was put on ciprofloxacin 500 mg oral tablets, two times daily, with metronidazole 400 mg oral tablets, three times daily, for a period of five days and later had no symptoms. The other patient developed a kind of surgical site infection, which resolved in a few days with an antibiotic prescription. Only one patient showed wound dehiscence. Resuturing was carried out after proper irrigation with betadine and saline. None of the patients showed any adverse reactions like allergies or darkening or discolouration of mucosa or skin.

Tests of significance

**Figure 7:**
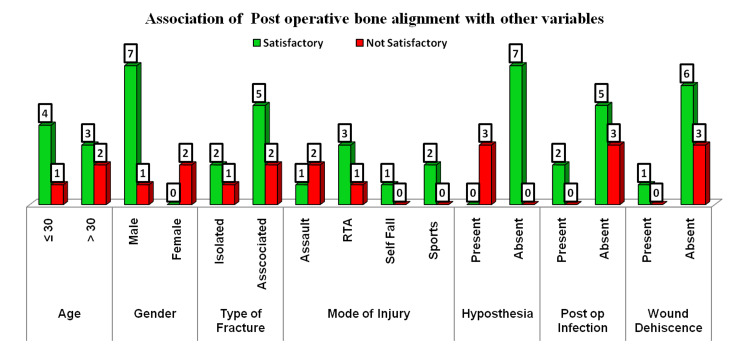
Association of postoperative bone alignment with other variables

The association of bone alignment with hypoesthesia or paresthesia revealed a highly significant p-value (0.002) using the Chi-square test. It is to be noted that postoperative infection and wound dehiscence were not significant with postoperative bone alignment (Figure 7 and Table 1).

**Table 1:** Association of postoperative bone alignment with other variables RTA: road traffic accident

Parameters		Bone alignment		Chi-square value	p-value
		Satisfactory	Not Satisfactory		
Age	≤ 30	4 (80%)	1 (20%)	0.476	0.490
	> 30	3 (60%)	2 (40%)		
Gender	Male	7 (87.5%)	1 (12.5%)	5.833	0.016
	Female	0	2 (100%)		
Type of fracture	Isolated	2 (66.7%)	1 (33.33%)	0.023	0.880
	Associated	5 (71.4%)	2 (28.6%)		
Mode of injury	Assault	1 (33.3%)	2 (66.7%)	3.254	0.354
	RTA	3 (75%)	1 (25%)		
	Self-reported fall	1 (100%)	0		
	Sports	2 (100%)	0		
Hypoesthesia (six months)	Present	0	3 (100%)	10.000	0.002
	Absent	7 (100%)	0		
Postoperative infection	Present	2 (100%)	0	1.071	0.301
	Absent	5 (62.5%)	3 (37.5%)		
Wound dehiscence	Present	1 (100%)	0	0.476	0.490
	Absent	6 (66.7%)	3 (33.3%)		

**Figure 8:**
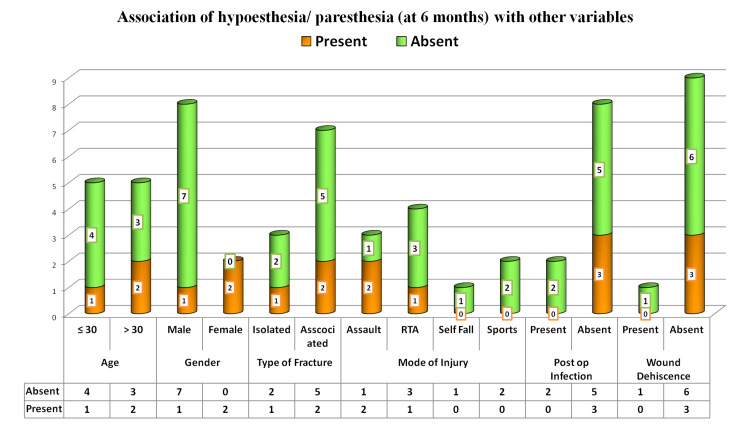
Association of six-month postoperative hypoesthesia or paresthesia with other variables

The association of hypoesthesia or paresthesia with postoperative infection and wound dehiscence showed insignificant results with a p-value greater than 0.05 (Figure 8 and Table 2).

**Table 2:** Association of six-month postoperative hypoesthesia or paresthesia with other variables

Parameters		Hypoesthesia or paresthesia		Chi-square value	p-value
		Present	Absent		
Age	≤ 30	1 (20%)	4 (80%)	0.476	0.490
	> 30	2 (40%)	3 (60%)		
Gender	Male	1 (12.5%)	7 (87.5%)	5.833	0.016
	Female	2 (100%)	0		
Type of fracture	Isolated	1 (33.3%)	2 (66.7%)	0.023	0.880
	Associated	2 (28.6%)	5 (71.4%)		
Mode of injury	Assault	2 (66.7%)	1 (33.3%)	3.254	0.354
	RTA	1 (25%)	3 (75%)		
	Self-reported fall	0	1 (100%)		
	Sports	0	2 (100%)		
Postoperative infection	Present	0	2 (100%)	1.071	0.301
	Absent	3 (37.5%)	5 (62.5%)		
Wound dehiscence	Present	0	1 (100%)	0.476	0.490
	Absent	3 (33.3%)	6 (66.7%)		

**Figure 9:**
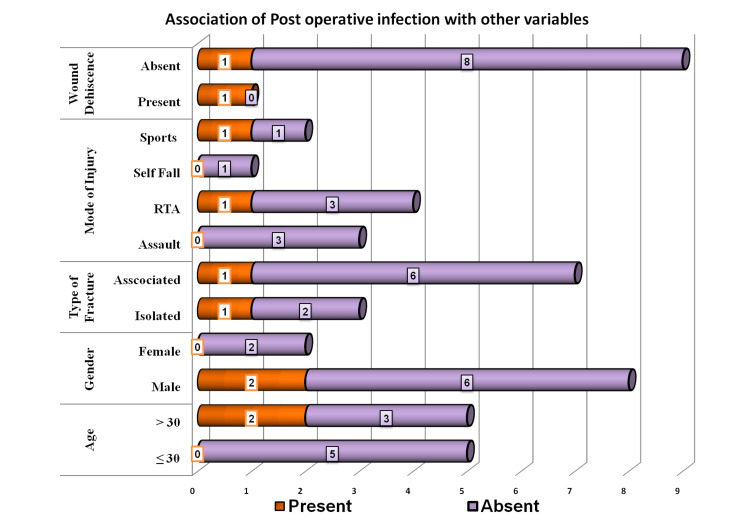
Association of postoperative infection with other variables

An association of postoperative infection was significant with the presence of wound dehiscence with a p-value of 0.035 (Figure 9 and Table 3).

**Table 3:** Association of postoperative infection with other variables

Parameter		Postoperative infection		Chi-square value	p-value
		Present	Absent		
Age	≤ 30	0	5 (100%)	2.500	0.114
	> 30	2 (40%)	3 (60%)		
Gender	Male	2 (25%)	6 (75%)	0.625	0.429
	Female	0	2 (100%)		
Type of fracture	Isolated	1 (33.3%)	2 (66.7%)	0.476	0.490
	Associated	1 (14.3%)	6 (85.7%)		
Mode of injury	Assault	0	3 (100%)	2.188	0.534
	RTA	1 (25%)	3 (75%)		
	Self-reported fall	0	1 (100%)		
	Sports	1 (50%)	1 (50%)		
Wound dehiscence	Present	1 (100%)	0	4.444	0.035
	Absent	1 (11.1%)	8 (88.9%)		

## Discussion

The maxillary sinus represents a weak area in the maxillofacial complex. Relevant bone fractures of the maxillary sinus can result from traumas with an anteroposterior vector or with an angular orientation. The traumatising energy spreads and meets virtually no resistance. This phenomenon explains why the fractures of the maxillary sinus anterior wall are marked by numerous comminuted fragments that are frequently displaced into the sinus itself.

According to studies by Maran et al. (1971), Anthony employed the first antral balloon via the intranasal route in 1952, which is how the treatment for treating comminuted fractures of the maxillary sinus was developed [4]. In this technique, the direct sublabial technique was avoided. The fragmented bone pieces were reduced by the pressure produced by inflating the balloon.

Maran et al. also said that in 1956, Jackson described two cases wherein they adopted the Shea-Anthony balloon catheter once more with the same approach in conjunction with external traction via Gillie’s temporal approach [4]. He also said that Jaraback used the 30 cm Foley catheter sublabially and transantrally after manual reduction, which was later modified by Gutman using intranasal antrostomy in 1965 via a sublabial transantral approach [4].

Other antral packing techniques include the use of ribbon-soaked gauze, most frequently in Whitehead's varnish. The removal of the pack frequently causes the patient discomfort and distress. A bone fragment on the margin of the intranasal antrostomy can rip the pack, which causes a portion of the pack to lodge inside the antrum and requires removal under general anaesthesia. If the pack is positioned sublabially, it might result in the development of the oro-antral fistula.

Zurbuchen recommended using split calvarial grafts or lyophilized cartilage layers to bridge the maxillary sinus wall defect in 1959. Biocompatibility was a key consideration in the use of allogenic lyophilized cartilage because 8.4% of patients experienced recurrent sinusitis [5].

According to Markus Zingg et al. (1992), anterior maxillary wall fragments that have become lodged in the sinus may need to be lifted, reduced, and fixed with resorbable sutures. The author also concluded that the miniplating of these fragments should be limited since it promotes fragment resorption and latent chronic sinusitis [5]. Thus, the need to implement a novel method to fix comminuted fracture segments using biocompatible alloplastic materials that are currently in use for other purposes was identified.

In 1949, Ardis discovered chemical adhesives. It was tested on humans after Coover reported on their adhesive capabilities in 1959 [6]. Polymethyl methacrylate (PMMA), cyanoacrylates, epoxy resins, polyurethanes, and calcium/magnesium phosphate types of cement are examples of synthetic adhesives that have been investigated for human use. These adhesives are borrowed from other industries or uses. Although they exhibit relatively poor biocompatibility and many of them are not biodegradable, they exhibit stronger bonding strength than biological adhesives. The cyanoacrylates are the group of adhesives that have received the most research. Numerous cyanoacrylates' properties, like adherence to bone and soft tissues under various storage settings and durations, have been documented in the literature.

Research done by Bhaskar et al. (1968) led to the application of cyanoacrylate tissue adhesives in dentistry. Rat tongues were divided in half for their tests and were subsequently joined with various adhesives. They found that butyl cyanoacrylate was the most well-tolerated by tissues, whereas methyl cyanoacrylate was the most cytotoxic. They also concluded that cyanoacrylate adhesives might be employed in the presence of moisture and as hemostatic agents [7].

Bhaskar and Frisch used butyl cyanoacrylate spray on 235 patients as a protective dressing over mucoperiosteal flaps, gingivectomies, painful ulcers, and extraction sites, allowing them to heal more quickly. Besserman in 1977 used butyl cyanoacrylate spray to stop bleeding from extraction sockets in patients with bleeding diatheses [8].

Weiko et al. were the first to use cyanoacrylate in human hard tissue in 1980 in four cases of anterior wall frontal sinus fracture. He found it relatively easy to use, with no adverse reactions. There was no interference with wound healing. The results observed by the authors were satisfactory. Though the parameters studied by these authors were sufficient, the study sample was quite small [9].

When PMMA bone cement and n-butyl-2-cyanoacrylate were used to fix the bone in an in vivo distal femoral osteotomy model in rabbits, Vihtonen et al. (1986) discovered that the combination provided enough stability for up to six weeks but that subsequent fixing was insufficient for effective healing [10].

In ten cases of mandibular fractures, Mehta et al. (1987) tried osteosynthesis using butyl cyanoacrylate. In the study, there were no plates or screws. The intermaxillary fixation (IMF) was only kept in place for 48-72 hours. When moderate manual force was applied to fracture segments, the authors observed no mobility. Radiographs taken after surgery showed no displacement of the broken segments. Analysis of blood, urine, and serum pre- and postoperatively showed no appreciable differences. After surgery, a chromosomal investigation found no increase in the incidence of sister-chromatid exchange (SCE) [11].

Gosain et al. (1998) compared the biomechanical strengths and concluded that titanium plates were the strongest form of fixation tested in both distraction and compression across a central gap. Also, resorbable plates fixed with bone adhesive were as strong as titanium plates; however, in distraction, fixation with bone adhesive, either alone or with resorbable plates, was weaker than a non-resorbable system [12].

Shermack et al. (1998) studied extensively the effects of cyanoacrylate in a rabbit craniotomy model. He studied the inflammatory responses of adjacent tissues in the scalp, cranium, and brain. Microdensitometric analysis of the bone gap revealed equal bone density in the cyanoacrylate and plating groups. Neurotoxicity analysis on the third and eleventh-week recovery periods revealed no inflammatory response [13].

In the same year, Shermack et al. reported on butyl cyanoacrylate fixation in the angle of mandibular osteotomies performed on rabbits and concluded that the bone adhesive fixation does not offer the biomechanical stability afforded by plates and screws in bones that are subjected to large forces [2].

Comprehensive research on tissue glues was conducted by Arun K Gosain (2002), and he concluded that n-butyl-2-cyanoacrylate held the most potential for fixing comminuted bone pieces. In parts of the facial skeleton where there are numerous tiny fragments of cortical bone, it proved to be a satisfactory alternative to other techniques of fixing [14].

Enrico Foresta et al. (2015) studied the application of n-butyl-2-cyanoacrylate to the anterior wall of maxillary sinus comminuted fractures in 25 patients. Both extraoral and intraoral approaches were used. There were no reports of hypoesthesia after a period of six months. No cases of displacement of fracture segments were seen, and no pseudoarthrosis was found [15].

In our study, we tried to find degrees of association between various variables and factors that could actually help in successful postoperative bone alignment and related morbidity, which was not attempted in previous studies. In our statistical analysis, we found that the association of hypoesthesia and paresthesia was highly significant with the level of bone alignment achieved (p-value = 0.002). There was also a high degree of association between gender and bone alignment (p-value of 0.016), but this may not be useful for the study because there were more males and very few females in the study group. Parameters like wound dehiscence and post-op infection were not related to the level of hypoesthesia.

There was a high degree of association between postop infection and wound dehiscence (p-value of 0.035). Since there were no adverse reactions in any of the cases, a statistical analysis could not be computed for wound dehiscence.

Limitations

The limitations of our study include the fact that the sample studied was smaller owing to the voluntary nature of participation in the study. Also, there was no control group in the study.

## Conclusions

The present study evaluated the outcome of n-butyl-2-cyanoacrylate tissue adhesive on hard tissue in non-load-bearing areas, in particular with comminuted fracture segments of the anterior wall of the maxillary sinus. Favourable bone alignment was seen in 70% of the patients. The n-butyl-2-cyanoacrylate application had no adverse reactions. Postoperatively, the favourable bone alignment had a direct influence on the regression of paresthesia innervated by the infraorbital nerve. Wound dehiscence had a direct correlation with postoperative infection. Thus, the bone adhesive can be safely applied over the comminuted fracture segments in any part of the face except the load-bearing vertical and horizontal pillars, where the masticatory forces are the highest. Further studies with a higher level of evidence and a larger sample size are needed to validate the usage of adhesives for bone fixation in comminuted fractures of dentate segments in the maxilla and mandible, along with mini plates to simplify the comminuted fracture into a single fracture line.
